# Preditores Pré-Operatórios de Readmissão Hospitalar em até 5 Anos após CRM: Análise de Coorte do Banco de Dados REPLICCAR II

**DOI:** 10.36660/abc.20240420

**Published:** 2025-03-06

**Authors:** Carlos Alberto Sancio, Fabiane Letícia de Freitas, Gabrielle Barbosa Borgomoni, Daniella de Lima Pes, Pedro Horigoshi Reis, Pedro Gabriel Melo de Barros e Silva, Marcelo Arruda Nakazone, Marcos Gradim Tiveron, Valquiria Pelisser Campagnucci, Luiz Augusto Lisboa, Luís Alberto Oliveira Dallan, Fabio Biscegli Jatene, Omar Asdrúbal Vilca Mejia

**Affiliations:** 1 Hospital Santa Rita de Cássia Vitoria ES Brasil Hospital Santa Rita de Cássia, Vitoria, ES – Brasil; 2 Hospital das Clínicas Faculdade de Medicina Universidade de São Paulo São Paulo SP Brasil Instituto do Coração do Hospital das Clínicas da Faculdade de Medicina da Universidade de São Paulo, São Paulo, SP – Brasil; 3 Hospital Samaritano Paulista São Paulo SP Brasil Hospital Samaritano Paulista, São Paulo, SP – Brasil; 4 Faculdade de Medicina de São José do Rio Preto São José do Rio Preto SP Brasil Faculdade de Medicina de São José do Rio Preto, São José do Rio Preto, SP – Brasil; 5 Irmandade da Santa Casa de Misericórdia de Marília Marilia SP Brasil Irmandade da Santa Casa de Misericórdia de Marília, Marilia, SP – Brasil; 6 Faculdade de Ciências Médicas Santa Casa de São Paulo São Paulo SP Brasil Faculdade de Ciências Médicas da Santa Casa de São Paulo, São Paulo, SP – Brasil

**Keywords:** Doença Arterial Coronariana, Revascularização Miocárdica, Hospitalização

## Abstract

**Fundamento:**

Reduzir as readmissões hospitalares após cirurgias de revascularização miocárdica (CRM) é essencial para otimizar os resultados a médio e longo prazo.

**Objetivo:**

Analisar preditores pré-operatórios associados à readmissão por todas as causas e cardíacas em até 5 anos após CRM.

**Métodos:**

Foram analisados 1387 pacientes submetidos à CRM entre junho de 2017 e julho de 2019, utilizando dados do registro multicêntrico REPLICCAR II. O seguimento foi realizado por entrevista telefônica com questionário estruturado no REDCap. A análise estatística incluiu métodos univariados e multivariados, utilizando regressão de Cox e validação interna do modelo por testes de calibração e discriminação. O nível de significância adotado foi de 5%.

**Resultados:**

A incidência cumulativa de readmissão por todas as causas foi de 27,69%, com um seguimento médio de 4,3 anos e tempo médio até a readmissão de 2,4 anos. A regressão multivariada indicou que menor índice de massa corporal (HR=0,97, p=0,032), histórico de infarto do miocárdio (HR=1,27, p=0,024), diabetes mellitus (HR=1,35, p=0,004), insuficiência renal (HR=1,62, p=0,004) e maior score STS (HR=1,22, p<0,001) estão associados a maior risco de readmissão por todas as causas. Foi observada correlação moderada entre readmissão e mortalidade (Rho=0,55).

**Conclusões:**

Esta análise revela que um índice de massa corporal mais baixo, antecedentes de infarto do miocárdio, diabetes mellitus, insuficiência renal e um STS score elevado estão associados ao aumento do risco de readmissão hospitalar após a CRM.

## Graphical abstract

**Figura Central:**
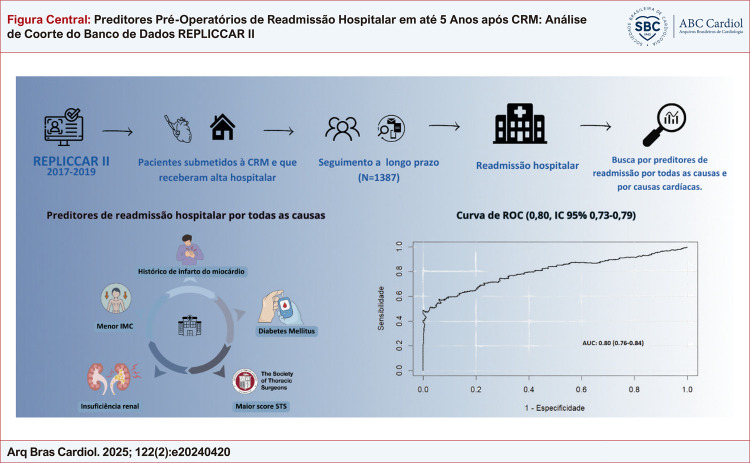
Preditores Pré-Operatórios de Readmissão Hospitalar em até 5 Anos após CRM: Análise de Coorte do Banco de Dados REPLICCAR II

## Introdução

O avanço das técnicas em cirurgia de revascularização do miocárdio (CRM), juntamente com a implementação de programas de qualidade nos hospitais, contribuíram significativamente para a redução das taxas de morbidade e mortalidade pós-operatórias.^1 , 2^ No entanto, apesar desses avanços, enfrentamos desafios com a readmissão hospitalar seja em curto prazo^3 - 5^ ou em longo prazo.^6 - 8^ Sendo esta última, mais difícil de definir pela falta de seguimento dos pacientes.

Portanto, a literatura médica recente tem concentrado sua atenção nas readmissões hospitalares que ocorrem em períodos mais curtos após a cirurgia cardíaca, especificamente dentro de 30 ou até 90 dias do procedimento. No entanto, é importante notar que os dados disponíveis sobre readmissões em longo prazo, ou seja, anos após a CRM, ainda são limitados e no nosso cenário estes resultados ainda não foram publicados pela falta de registros em longo prazo. Informações que são fundamentais para a definição de estratégias custo-eficazes nas CRM.^9^

Em uma subanálise do “ *CORONARY trial”,* que estudou as causas de readmissão hospitalar em um período de cinco anos após CRM identificaram que ser mulher, idoso, apresentar índices elevados de massa corporal, histórico de infarto agudo do miocárdio, acidente vascular cerebral prévio, doença arterial periférica, tabagismo ativo e diabetes mellitus se associaram a um risco aumentado de readmissão hospitalar por todas as causas.^7^

Identificar estes fatores de risco ajudaria na estratificação de grupos de risco com a finalidade de prevenção, assim como de melhorar a indicação e, principalmente, a eficácia das CRM.^10^ Um assunto importante para a geração de valor e redução dos custos no sistema de saúde por complicações.^10^ Na literatura existe uma diversidade de abordagens metodológicas, incluindo amostras de tamanhos variados, distintos critérios para alocar os pacientes e desfechos com variados graus de relevância para a readmissão hospitalar, ressaltando a necessidade de evidências mais sólidas relacionadas a este tópico.

Por conseguinte, torna-se importante identificar os fatores que contribuem para a ocorrência de readmissão hospitalar em médio e longo prazo, com o objetivo de otimizar os resultados das cirurgias cardíacas e minimizar os encargos financeiros para o sistema de saúde. Sendo assim,o objetivo deste estudo foi identificar os preditores de readmissão hospitalar por todas as causas e por causas cardíacas em até cinco anos após a CRM.

## Métodos

Este é um estudo a partir dos dados do Registro Paulista de Cirurgia Cardiovascular II (REPLICCAR II), um estudo prospectivo, multicêntrico (incluindo 5 hospitais do estado de São Paulo), conduzido com pacientes submetidos à cirurgia de revascularização miocárdica.

O banco de dados REPLICCAR II contém pacientes com idade ≥18 anos, submetidos à CRM primária e isolada de forma eletiva ou urgência. A plataforma para coleta de dados foi criada no REDCap (http://www.project-redcap.org) especialmente para o projeto, na qual foi feita a recolecção de forma online por profissionais graduados e treinados. O banco de dados contém as mesmas variáveis e definições da versão 2.9 do sistema de coletas do STS ( *Society of Thoracic Surgeons* ).^11^ Foram excluídos da análise os pacientes que faleceram antes da alta hospitalar, os readmitidos dentro de 30 dias após a alta e aqueles que não realizaram a CRM como um procedimento isolado. De forma que este estudo foca na readmissão ocorrida mais de 30 dias após a alta.

### Seguimento dos pacientes

**Figura 1:**
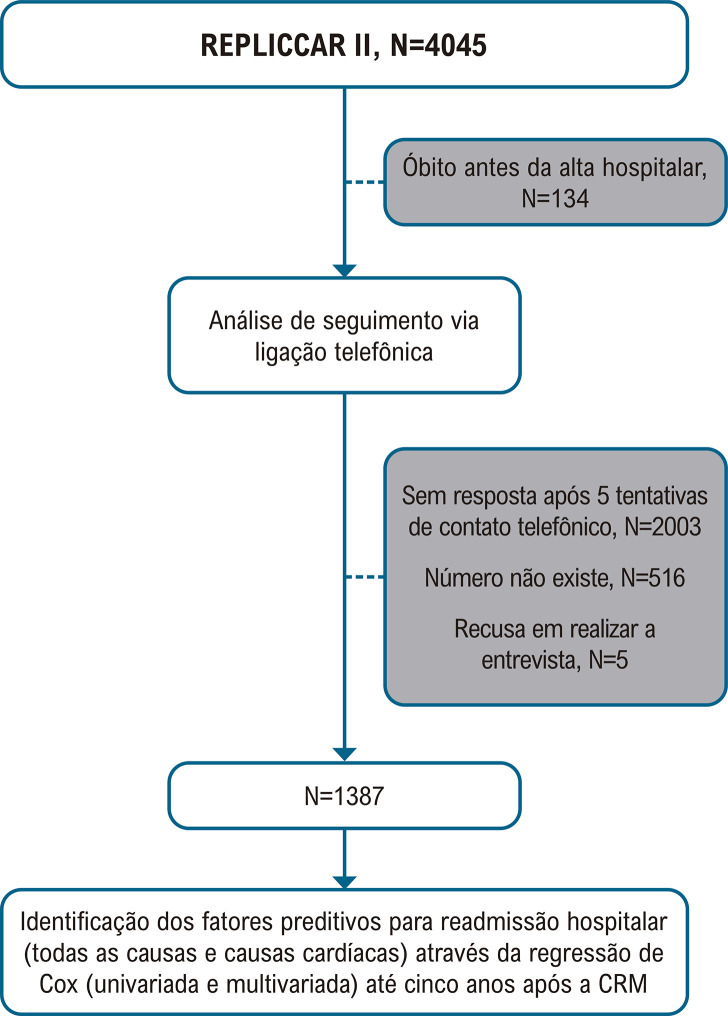
– Fluxograma da análise. REPLICCAR II: Registro Paulista de Cirurgia Cardiovascular; CRM: revascularização miocárdica. Os autores seguiram os critérios estabelecidos pelo STROCSS. ^12^

Os dados relacionados ao seguimento foram coletados de abril de 2023 a janeiro de 2024, os pacientes responderam um questionário estruturado relacionado à qualidade de vida, sintomas cardíacos, ocorrência de eventos adversos cardíacos maiores e readmissões hospitalares (N=384), como representado na Figura 1 a seguir.

### Definição das variáveis

A variável de desfecho primário analisado neste estudo foi a readmissão hospitalar por todas as causas. Definimos a readmissão hospitalar como o retorno do paciente ao hospital dentro de um período de até cinco anos após a realização da CRM.

O desfecho secundário foi a readmissão hospitalar não planejada relacionada a causas cardíacas, definida como insuficiência cardíaca, arritmias, angina, reoperação de CRM ou intervenção coronária percutânea.

### Análise estatística

Para todas as análises deste estudo, utilizou-se o software R, versão 4.0.2,^13^ para este projeto, recorreu-se aos pacotes *survival, car, survminer, psych, gmodels, survivalROC, timeROC, pROC, resource selection, ggplot2 e dplyr.*

Na análise descritiva, as variáveis contínuas foram expressas exclusivamente pela mediana e o intervalo interquartil (IIQ), devido à distribuição assimétrica dos dados. As variáveis categóricas foram apresentadas em termos de frequências e porcentagens.

Para a análise das variáveis independentes categóricas, realizamos a comparação de proporções utilizando o teste qui-quadrado ou o teste exato de Fisher, conforme adequado. A normalidade dos dados foi verificada através do teste de Shapiro-Wilk. Para as variáveis independentes contínuas e o desfecho do estudo, utilizamos o teste de Mann-Whitney para a comparação de médias, uma vez que todas as variáveis apresentaram distribuição não paramétrica. A análise estatística das variáveis preditivas, abrangendo tanto fatores pré-operatórios quanto intraoperatórios, iniciou-se com a aplicação da regressão logística univariada de *Cox* . Esta etapa preliminar visou a identificação de variáveis com valores de p<0,05, que foram posteriormente incorporadas ao modelo de regressão multivariada de Cox para uma análise mais aprofundada. Os achados foram apresentados em termos de razão de risco (HR: Hazard ratio) acompanhada dos respectivos intervalos de confiança de 95% (IC 95%).

Para avaliar o modelo múltiplo, realizamos o teste de resíduos de *Schoenfeld* , adequado para a regressão de *Cox* , além da análise da curva *Receiver Operating Characteristic* (ROC).

A correlação entre a readmissão hospitalar por todas as causas e a mortalidade foi avaliada utilizando a correlação de Spearman. Os valores de Rho foram interpretados da seguinte forma: Rho = 0 indica ausência de correlação; 0 < ∣Rho∣ ≤ 0,3 indica correlação fraca; 0,3 < ∣Rho∣ ≤ 0,7 indica correlação moderada; e ∣Rho∣ > 0,7 indica correlação forte. O nível de significância adotado foi de 5%.

### Ética e consentimento

Este estudo é uma subanálise do projeto REPLICCAR II, aprovado pelo Comitê de Ética em Pesquisa para a Análise de Projetos (CAPPesq) do Hospital das Clínicas da Universidade de São Paulo (número de registro CAAE: 66919417.6.1001.0068; SDC 4506/17/006). Para a fase de acompanhamento, uma emenda (parecer número 5.603.742) foi aprovada em 25 de agosto de 2022. Todos os pacientes forneceram consentimento para participar da entrevista.

## Resultados

Na comparação entre pacientes não readmitidos e readmitidos por todas as causas ( Tabela 1 ), observou-se que o grupo readmitido apresentou um índice de massa corporal ligeiramente menor (p=0,022). Além disso, a incidência de infarto prévio do miocárdio foi significativamente maior nesse grupo (p=0,004), assim como a prevalência de diabetes mellitus (p=0,002). Notou-se também uma proporção significativamente maior de pacientes insulinodependentes entre os readmitidos (p<0,001). Quanto às condições de saúde, o grupo readmitido teve maior frequência de doença cerebrovascular (p=0,001) e insuficiência renal (p<0,001). Este grupo também registrou níveis de creatinina mais elevados, indicando diferenças significativas (p<0,001). A análise da função cardíaca revelou que uma fração de ejeção abaixo de 30% foi mais comum entre os readmitidos (p=0,039). Em termos de classificação funcional, pacientes com uma classificação NYHA III e IV foram mais prevalentes no grupo readmitido (p=0,006), sugerindo uma maior severidade de insuficiência cardíaca. Por fim, o STS score para mortalidade também foi maior no grupo de pacientes readmitidos (p<0,001).

**Tabela 1:** – Características pré-operatórias dos pacientes submetidos à CRM. REPLICCAR II, São Paulo – Brasil

Características	Não readmitido (N=1003)	Readmitido (N=384)	Valor de p
**Idade (anos), (mediana e IIQ)**	64 (57-70)	64 (59-71)	0,118
**Sexo feminino, n (%)**	256 (25,52)	107 (27,86)	0,375
**Status de admissão, n (%)**			
Eletivo	574 (57,23)	198 (51,56)	
Urgência/Emergência	221 (22,03)	105 (27,34)	0,086
Transferência de outro hospital	201 (20,04)	81 (21,09)	
Outro	4 (0,40)	0 (0,00)	
**Índice de massa corporal, kg/m^2^ (mediana e IIQ)**	27 (24,50-29,76)	26,57 (24,22-29,38)	0,022
**Infarto prévio do miocárdio, n (%)**	495 (49,35)	223 (58,07)	0,004
**Hipertensão arterial sistêmica, n (%)**	880 (87,74)	343 (89,32)	0,413
**Doença pulmonar, n (%)**			
Leve	10 (1,00)	6 (1,56)	0,321
Moderada	4 (0,40)	3 (0,78)	
Severa	5 (0,50)	0 (0,00)	
**Tabagismo, n (%)**			
Nunca	509 (50,75)	185 (48,16)	0,549
Fumante ativo	146 (14,56)	64 (16,67)	
Ex-fumante	348 (34,70)	135 (35,16)	
**Diabetes mellitus, n (%)**	484 (48,26)	221 (57,55)	0,002
Insulinodependente	114 (11,37)	71 (18,49)	< 0,001
**Doença cerebrovascular, n (%)**	79 (7,88)	52 (13,54)	0,001
**Insuficiência renal, n (%)**			
Crônica	42 (4,19)	40 (10,42)	<0,001
Aguda	7 (0,70)	3 (0,78)	
**Creatinina, mg/Dl (mediana e IIQ)**	1,07 (0,93-1,37)	1,16 (0,90-1,23)	< 0,001
**Angioplastia prévia, n (%)**	134 (13,36)	63 (16,41)	0,145
**Fração de ejeção (<30%), n (%)**	11 (1,10)	10 (2,60)	0,039
**CCS, n (%)**			
IV	89 (8,87)	40 (10,42)	0,376
**NYHA, n (%)**			
I e II	891 (88,83)	316	0,006
III e IV	112 (11,17)	68	
**STS score de mortalidade, (mediana e IIQ)**	0,81 (0,43-0,97)	1,05	<0,001

IIQ: intervalo interquartil CCS: Classificação da Sociedade Cardiovascular Canadense de angina; NYHA: Classificação funcional da New York Heart Association; STS: Society of Thoracic Surgeons; Doença cerebrovascular: Acidente Vascular Cerebral, Ataque Isquêmico Transitório ou Estenose das Carótidas maior ou igual a 50%; Insuficiência renal: Considerado Clearance de creatinina <60 ml/min/1,73m^2^.

Houve diferenças significativas entre os pacientes não readmitidos e readmitidos em relação ao uso da circulação extracorpórea e à taxa de extubação na sala cirúrgica (p=0,028 e 0,040, respectivamente). Adicionalmente, os níveis de glicemia foram significativamente mais altos (p<0,001) nos pacientes readmitidos, conforme detalhado na tabela 2 .

**Tabela 2:** – Características intraoperatória dos pacientes submetidos à CRM. REPLICCAR II, São Paulo – Brasil

Características	Não readmitido (N=1003)	Readmitido (N=384)	Valor de p
**Utilização de circulação extracorpórea, n (%)**	901 (89,93)	328 (85,42)	0,028
**Tempo de circulação extracorpórea (minutos), (mediana e IIQ)**	75 (59,00-95,25)	75 (56-100)	0,998
**Tempo de anoxia (minutos), (mediana e IIQ)**	57 (43-75)	59 (41-79)	0,516
**Uso da artéria torácica bilateral, n (%)**	135 (13,46)	45 (11,72)	0,388
**Uso da artéria torácica interna esquerda, n (%)**	966 (96,31)	361 (94,01)	0,059
Pediculada	629 (65,11)	243 (67,31)	0,128
Esqueletizada	337 (34,89)	118 (32,69)	
**Uso da artéria torácica interna direita, n (%)**	145 (14,46)	51 (13,28)	0,573
Pediculada	75 (51,72)	27 (52,94)	0,844
Esqueletizada	70 (48,28)	24 (47,06)	
**Uso de artéria radial, n (%)**	46 (4,59)	18 (4,69)	0,935
**Tempo de cirurgia (horas), (mediana e IIQ)**	4,50 (3,42-6,00)	4,67 (3,50-6,08)	0,314
**Transfusão de concentrados de hemácias, n (%)**	8 (0,80)	3 (0,78)	0,970
**Extubação em sala cirúrgica, n (%)**	48 (4,79)	9 (2,34)	0,040
**Glicemia mais alta, (mediana e IIQ)**	174 (47,17-93,08)	177 (50,50-115,25)	< 0,001

IIQ: intervalo interquartil.

Entre os pacientes analisados na tabela 3 , os readmitidos apresentaram uma maior incidência de insuficiência renal (p<0,001), maior taxa de reoperação por sangramento (p=0,009), e maior tempo de intubação orotraqueal (p=0,001). Além disso, a ventilação prolongada por mais de 24 horas (p<0,001) e a ocorrência de infecção da ferida operatória (p=0,013) foram mais comuns entre os readmitidos. Esses pacientes também tiveram internações hospitalares (p<0,001) e permanências na UTI mais longas (p<0,001), incluindo internações hospitalares com mais de 14 dias (p<0,001), destacando desafios adicionais no manejo pós-operatório dos pacientes readmitidos.

**Tabela 3:** – Variáveis de desfecho e evolução pós-operatória dos pacientes submetidos à CRM. REPLICCAR II, São Paulo – Brasil

Características	Não readmitido (N=1003)	Readmitido (N=384)	Valor de p
**Acidente Vascular Cerebral, n (%)**	13 (1,30)	5 (1,30)	0,992
**Insuficiência Renal, n (%)**	45 (4,49)	46 (11,98)	< 0,001
**Reoperação por sangramento, n (%)**	2 (0,20)	5 (1,30)	0,009
**Fibrilação atrial, n (%)**	143 (14,26)	68 (17,71)	0,109
**Tempo intubação orotraqueal (horas), (mediana e IIQ)**	7,78 (5,33-11,01)	8,5 (6,04-12,23)	0,001
**Ventilação prolongada (>24 horas), n (%)**	25 (2,49)	24 (6,25)	< 0,001
**Infecção ferida operatória (≤30 dias), n (%)**	22 (2,19)	18 (4,69)	0,013
**Internação hospitalar prolongada (>14 dias), n (%)**	222 (22,13)	119 (30,99)	< 0,001
**Internação hospitalar curta (<6 dias), n (%)**	52 (5,18)	13 (3,39)	0,156
**Tempo de permanência na UTI (horas), (mediana e IIQ)**	68,12 (47,17-93,08)	73,17 (50,50-115,25)	< 0,001
**Tempo de permanência hospitalar (dias), (mediana e IIQ)**	7,00 (6,00-8,00)	7,00 (6,00-10,00)	< 0,001
**Óbito em 30 dias após o procedimento, n (%)**	0 (0,00)	5 (1,30)	0,002

IIQ: intervalo interquartil.

Para identificação dos fatores associados ao desfecho de readmissão por todas as causas, a regressão univariada foi utilizada para identificar variáveis correlacionadas ao evento ( Tabela suplementar 1 ). Em seguida, a partir dos dados obtidos na análise univariada, a regressão multivariada foi utilizada para criação de um modelo múltiplo ( Tabela 4 ).

**Tabela 4:** – Estimativas da associação entre características do paciente e readmissão por todas as causas usando um modelo de regressão multivariada de Cox ajustado para características dos pacientes. REPLICCAR II, São Paulo – Brasil

Características	OR	IC 95%	Valor de P
**Status de admissão**			
Urgência	1,11	0,99 - 1,24	0,070
**Índice de massa corporal, kg/m^2^**	0,97	0,94 - 0,99	0,029
**Infarto prévio do miocárdio**	1,27	1,03 - 1,56	0,024
**Diabetes mellitus**	1,35	1,10 - 1,66	0,004
**Insuficiência renal**	1,62	1,16 - 2,25	0,004
**Fração de ejeção (<30%)**	1,32	0,70 - 2,50	0,394
**NYHA**			
III e IV	1,28	0,97 - 1,67	0,077
**STS score (mortalidade)**	1,22	1,09 - 1,36	< 0,001

NYHA: Classificação funcional da New York Heart Association; STS: Society of Thoracic Surgeons; A: Considerado clearance de creatinina < 60 ml/min/1,73m^2^; HR: Hazard ratio; IC 95%: Intervalo de Confiança de 95%.

**Figura 2:**
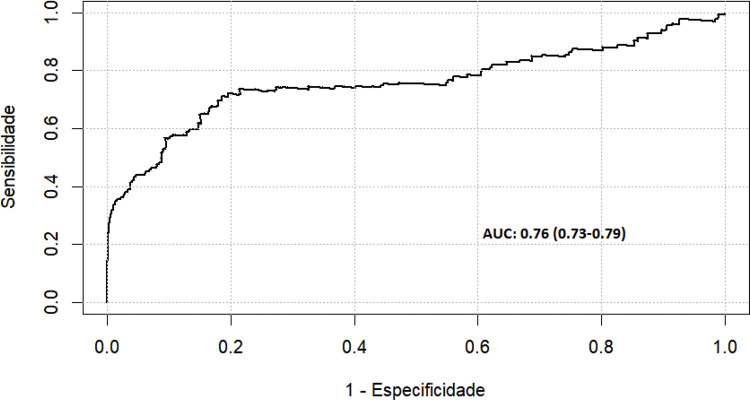
– Curva ROC do modelo de regressão multivariada de Cox ajustado para os pacientes que foram readmitidos por todas as causas.

Para validação do modelo foi realizado o teste de Resíduos de Schoenfeld, os resultados do teste não revelaram evidências significativas de violações da suposição de riscos proporcionais, ou seja, o modelo estava calibrado (p=0,192). Além disso, a curva de ROC (0,80, IC 95% 0,73-0,79) demonstrou que o modelo múltiplo é preciso para prever readmissão por todas as causas em até 5 anos após a CRM ( Figura 2 ) ( Figura Central ).

Para identificação dos fatores associados ao desfecho de readmissão por causas cardíacas, a regressão univariada foi utilizada para identificar variáveis correlacionadas ao evento ( Tabela Suplementar 2 ). Em seguida, a partir dos dados obtidos na análise univariada, a regressão multivariada foi utilizada para criação de um modelo múltiplo ( Tabela 5 ).

**Tabela 5:** – Estimativas da associação entre características do paciente e readmissão por causas cardíacas usando um modelo de regressão multivariada de Cox ajustado para características dos pacientes. REPLICCAR II, São Paulo – Brasil

Características	HR	IC 95%	Valor de p
**Status de admissão**			
Urgência	1,22	1,02 - 1,46	0,026
**Índice de massa corporal, kg/m^2^**	0,94	0,90 - 0,98	0,005
**Fração de ejeção (<30%)**	3,15	1,47 - 6,76	0,003

HR: Hazard ratio; IC 95%: Intervalo de Confiança de 95%.

**Figura 3:**
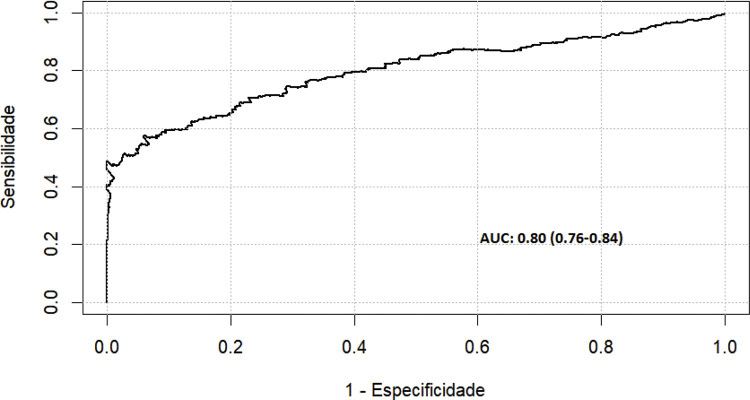
– Curva ROC do modelo de regressão multivariada de Cox ajustado para os pacientes que foram readmitidos por causas cardíacas.

Para validação do modelo foi realizado o teste de Resíduos de Schoenfeld, os resultados do teste não revelaram evidências significativas de violações da suposição de riscos proporcionais, ou seja, o modelo estava calibrado (p=0,40). Além disso, a curva de ROC (0,76, IC 95% 0,73-0,79) ( Figura 3 ) demonstrou que o modelo múltiplo é preciso para prever readmissão por causas cardíacas em até 5 anos após a CRM.

**Figura 4:**
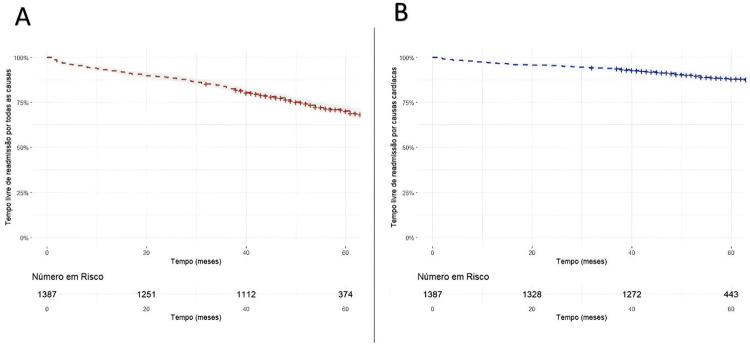
– Incidência cumulativa de readmissão por todas as causas (A) e cardíaca (B) na coorte geral durante o seguimento.

A média de seguimento dos pacientes foi de 4,3 anos (percentil 25 e 75: 3,5-5,0), a incidência cumulativa de readmissão por todas as causas foi de 27,69% (IC 95% 0,25 – 0,30) como representado na Figura 4-A. O tempo médio de readmissão por todas as causas foi de 2,4 anos (percentil 25 e 75: 1,0-3,6). A incidência cumulativa de readmissão por causas cardíacas foi de 10,67% (IC 95%: Em relação a readmissão por causa cardíaca (Figura 4-B), o tempo médio foi de 2,33 anos (percentil 25 e 75: 0,75-3,69).

Entre os pacientes que não foram readmitidos, apenas 15 (1,50%) evoluíram para óbito. Em contraste, dentre os pacientes readmitidos, 154 (40,10%) evoluíram para óbito. Foi observada uma correlação moderada entre a readmissão hospitalar por todas as causas e a ocorrência de óbito, apresentando um coeficiente Rho de 0,55 (IC 95%, 0,51-0,59).

## Discussão

Nesta análise de seguimento de médio e longo prazo em pacientes submetidos a CRM no estado de São Paulo por meio de dados multicêntricos, foi identificado que a média de incidência cumulativa de readmissão por causas cardíacas e todas as causas foram de 10,67% e de 27,69%, respectivamente. Estando esta última correlacionada com a mortalidade, em conformidade com a literatura atual.^7^

Dentre os preditores identificados, o status cirúrgico de urgência, o índice de massa corporal (IMC) reduzido e a fração de ejeção do ventrículo esquerdo (FEVE) <30% foram relevantes para ambos os desfechos avaliados, refletindo também a complexidade dos pacientes em nosso cenário.

Apesar de a mortalidade em cirurgia cardíaca ter diminuído significativamente a partir do refinamento das técnicas cirúrgicas, maquinários, linha de cuidados, bancos de dados e outros,^2 , 14 , 15^atualmente a atenção aos desfechos não relacionados ao óbito tem ganhado espaço nas discussões no âmbito da qualidade e segurança do paciente, entretanto, estes dados ainda são raros e dispersos em países de baixa e média renda, sobretudo em relação ao acompanhamento a longo prazo, onde o foco nas análises permeia os primeiros 30 a 90 dias após a alta, apresentando uma heterogeneidade de taxas entre 8,3 e 21,1% entre as análises.^4 , 16 , 17^

A ausência de grandes bancos de dados em saúde, devidamente estruturados para o seguimento a longo prazo, nos países em desenvolvimento não apenas limita a capacidade de realizar pesquisas robustas e o desenvolvimento de estratégias guiadas por dados, como também compromete o acompanhamento dos pacientes submetidos a CRM. Sem um registro detalhado, com atualizações periódicas, históricos médicos de diagnósticos e intervenções, os profissionais de saúde, gestores hospitalares e de saúde pública enfrentam obstáculos no monitoramento do progresso dos pacientes, na identificação de complicações pós-operatórias, na adaptação de planos de cuidados assistências, e na própria evolução do paciente. A implementação de sistemas de dados amigável e robusto é crucial para a formação de uma cultura baseada em dados, melhoria contínua dos cuidados em saúde, garantindo a identificação de doenças com maior agilidade, indicação do melhor tratamento e acompanhando sua evolução,^18^ como é o caso da readmissão hospitalar, desfecho que culmina em pior prognóstico e aumento de custos para o sistema de saúde.^19 , 20^

Em nossa análise, foi observado que 40,10% dos pacientes que necessitaram realizar ao menos uma reinternação após a alta hospitalar pós-CRM evoluíram para óbito. Da mesma forma, Bianco et al.^21^ avaliaram os impactos a longo prazo da readmissão após 30 dias da cirurgia (n=14538) e apontaram que a reinternação esteve significativamente associada à mortalidade tanto a curto (6 meses) quanto a longo seguimento (60 meses), mas também um preditor independente para novas readmissões. É necessário realizar mais estudos para avaliar a qualidade de vida dos pacientes que, após procedimentos cardíacos, necessitam de novas internações. Essa avaliação deve considerar não apenas aspectos clínicos, mas também psicossociais, permitindo que os hospitais desenvolvam ferramentas adequadas de acompanhamento e follow-up, além de estratégias para reduzir a probabilidade de readmissões. Neste cenário, a criação de modelos preditivos e escores surge como uma ferramenta poderosa para auxiliar nas decisões médicas e multiprofissionais, com o potencial de reduzir significativamente o risco de readmissão. Os fatores associados à readmissão apontados pela regressão múltipla refletem a complexidade do paciente no pré-operatório, e podem oportunizar janelas de melhorias, semelhantes aos achados da literatura.^6^ O recente ensaio *CORONARY trial* ^6^ avaliou 4623 pacientes com seguimento a longo prazo e destacou que as taxas de readmissão hospitalar, tanto para todas as causas quanto para cardíacas, foram significativas após 5 anos da alta hospitalar após CRM, onde mulheres apresentaram risco significativamente maior de readmissão, com uma correlação forte entre readmissão e mortalidade a longo prazo, evidenciando diferenças de gênero significativas no prognostico após o procedimento. Em nosso estudo, o sexo não teve associação em nenhum dos desfechos avaliados, entretanto, a readmissão por todas as causas foi correlacionada moderadamente com a mortalidade. Mais estudos precisam ser realizados a fim de explorar o impacto do sexo na evolução dos pacientes submetidos a CRM.^22^

Em nossa análise IMC baixo foi um preditor de readmissão hospitalar em longo prazo. A desnutrição, fator correlacionado à readmissão na nossa análise, pode estar vinculada à demora para a realização da cirurgia cardíaca devido à considerável fila cirúrgica no sistema público de saúde, sem o devido acompanhamento multiprofissional estimulando a pré-habilitação do paciente, mas também pode ter relação com a perda de peso observada durante o período de internação pré-cirurgia, sabendo-se que sarcopenia está associada a desfechos clínicos menos favoráveis.^23^ Este achado indica a importância da avaliação nutricional e do manejo adequado do peso do paciente desde a indicação cirúrgica, o preparando para o procedimento a partir de linhas de cuidados personalizadas e preventivas, como estratégias para otimizar o estado clínico do paciente no momento da cirurgia, promovendo uma recuperação mais eficiente e com potencial de minimizar o risco de readmissão.

A insuficiência cardíaca está sabidamente associada à necessidade de múltiplas internações e à piora de morbimortalidade.^24 - 26^ A identificação da FEVE <30% como preditor de readmissão hospitalar é justificável e pode ser explicada pela demora no diagnóstico da doença cardiovascular ou devido ao longo período de espera para o tratamento cirúrgico sendo esta realizada muitas vezes com caráter de urgência.^27^ A FEVE <30% pode levar ao deterioramento do estado clínico do paciente gerando descompensações que necessitam de novas internações.

O uso de circulação extracorpórea (CEC) foi menor entre os pacientes readmitidos. Estudos indicam que a CEC pode aumentar o risco de complicações devido à resposta inflamatória sistêmica e ao trauma causado pela máquina.^28^ No entanto, como este estudo não é randomizado, é possível que os pacientes que não utilizaram CEC tivessem um perfil clínico mais grave, como aorta em porcelana ou outros fatores de risco que não foram mensurados nesta análise. Isso pode ter influenciado os diferentes perfis de complicações observados. Embora a literatura sugira que cirurgias sem CEC podem reduzir complicações imediatas, ainda não há consenso sobre o impacto a longo prazo.^29^

A extubação em sala cirúrgica foi menos comum entre os pacientes readmitidos. Essa prática é geralmente associada a melhores desfechos, como redução da ventilação mecânica, do tempo de UTI e da duração da hospitalização.^30 , 31^ A literatura indica que a extubação precoce, quando realizada em pacientes devidamente selecionados, pode diminuir o risco de infecções pulmonares e promover uma recuperação pós-cirúrgica mais rápida, o que pode contribuir para uma menor taxa de readmissões.

Estudos mostram que um controle glicêmico rigoroso pode reduzir o risco de complicações pós-operatórias e readmissões hospitalares, especialmente em pacientes diabéticos. Em nosso estudo, observamos que os pacientes readmitidos apresentaram níveis glicêmicos mais elevados em comparação com os não readmitidos. A hiperglicemia no período perioperatório está associada a piores desfechos, incluindo aumento de complicações infecciosas, atraso na cicatrização e maior mortalidade. Esses achados estão alinhados com a literatura, que destaca a importância de manter um controle glicêmico adequado durante o período intraoperatório, independentemente de o paciente ser diabético ou não.^32 , 33^

Os pacientes readmitidos a longo prazo apresentaram características hospitalares associadas a piores desfechos, como insuficiência renal, maior taxa de reoperações por sangramento e infecção profunda/mediastinite. Esses achados indicam que complicações pós-operatórias, associadas a maior tempo de internação hospitalar e em UTI, estão relacionadas ao aumento das readmissões a longo prazo. A utilização de indicadores para avaliar o desempenho da nossa prática clínica é fundamental para o gerenciamento eficaz das instituições. A readmissão hospitalar não apenas reflete a qualidade do atendimento e a satisfação do paciente, mas também permite monitorar processos que podem levar a reinternações. Tais eventos postergam o retorno do paciente às suas atividades diárias, expõem-no novamente ao ambiente hospitalar, aumentam o risco de complicações e, potencialmente, elevam o risco de morte. Em nossa análise, destacamos a importância desse indicador, observando uma correlação moderada entre readmissão por todas as causas e mortalidade, com um coeficiente Rho de 0,55 (IC 95%: 0,51 a 0,59) ( Figura 4 ). A identificação e o monitoramento desses indicadores são cruciais para o desenvolvimento de estratégias proativas voltadas à melhoria dos resultados assistenciais e à redução de custos.^20^

Um dos principais desafios deste estudo foi o acompanhamento dos pacientes, que exigiu a realização de ligações telefônicas. O Brasil ainda carece de um sistema único de banco de dados que centralize informações sobre readmissões e óbitos, o que poderia facilitar e fazer avançar a pesquisa nacional. Enquanto não tivermos uma integração eficaz de dados, será difícil obter um seguimento completo e preciso dos pacientes. A ausência de um sistema unificado dificulta a coleta e análise de dados que são essenciais para a melhoria contínua da qualidade dos cuidados e da gestão em saúde.

Acreditamos que a implementação de um banco de dados com seguimento dos pacientes tenha potencial para identificar preditores e guiar intervenções, de forma que o atendimento médico cirúrgico possa melhorar resultados na qualidade de vida dos pacientes.

### Limitações do estudo

O presente estudo foi realizado com uma coorte preliminar de um banco de dados multicêntrico no estado de São Paulo. Os seguimentos continuam sendo coletados pelo grupo de estudos até o contato (ou 5ª tentativa) para cada paciente incluído no banco de dados primário. A dificuldade em localizar uma significativa proporção dos pacientes inicialmente incluídos no estudo pode comprometer a precisão e a generalização dos resultados obtidos. Os resultados por instituição podem ter refletido algum nível de impacto na análise devido a heterogeneidade relacionada à experiência das equipes de saúde, materiais disponíveis, metodologias e protocolos institucionais e até mesmo o perfil do paciente atendido por hospital de modo de transmitir um retrato de mundo-real. Portanto é recomendado que cada instituição avalie isoladamente seus dados para validação dos resultados aqui expostos e direcionamento de condutas para melhorias dos resultados. A descrição das complicações até 30 dias após CRM não foram objeto da presente análise, pois trata-se de um estudo com pacientes sobreviventes à cirurgia com o objetivo de compreender quais os preditores pré-operatórios que influenciaram na readmissão hospitalar e desta forma criar estratégias para diminuir este impacto. Esta é uma análise post-hoc, pois o seguimento a longo prazo não foi objetivo de análise principal do banco de dados REPLICCAR II, por isso é possível que esta analise não tenha poder estatístico suficiente para validar a hipótese, por esta razão recomendamos que mais estudos sejam realizados com esta finalidade.

## Conclusão

As variáveis pré-operatórias IMC baixo, infarto do miocárdio, diabetes mellitus, insuficiência renal e um STS score elevado se associaram ao aumento do risco de readmissão hospitalar 5 anos após a CRM. Assim mesmo, cirurgia na urgência, IMC baixo e fração de ejeção <30% foram preditores das readmissões por causas cardíacas.
