# Supplementary material for: Preditores Pré-Operatórios de Readmissão Hospitalar em até 5 Anos após CRM: Análise de Coorte do Banco de Dados REPLICCAR II
Source: Arq Bras Cardiol. 2025 Mar 6;122(2):e20240420. [Article in Portuguese] doi: 10.36660/abc.20240420 (PMC12080614; doi:10.36660/abc.20240420)
Supplement: Supplementary file 1 [file 0066-782X-abc-122-2-e20240420-suppl01.pdf]

SUPLEMENTAR

Tabela 1. Estimativas da associação entre características do paciente e readmissão por todas as causas usando um modelo de regressão univariada de Cox para a seleção de variáveis para o modelo múltiplo.

Características	HR	IC 95%	Valor de *P*
**Idade**	1,00	1,00 - 1,02	0,123
**Sexo (feminino)**	1,11	0,89 - 1,38	0,365
**Status de admissão**			
Urgência/Emergência	1,40	1,12 - 1,76	0,003
**Índice de massa corporal, kg/m²**	0,97	0,94 - 0,99	0,008
**Infarto prévio do miocárdio**	1,36	1,11 - 1,66	0,003
**Hipertensão arterial sistêmica**	1,14	0,63 - 1,21	0,436
**Doença pulmonar**	1,13	0,59 - 2,20	0,703
**Tabagismo**			
Nunca	Referência	Referência	
Fumante ativo	1,18	0,88 - 1,57	0,626
Ex-fumante	1,05	0,85 - 1,32	0,255
**Diabetes mellitus**	1,41	1,15 - 1,72	0,001
**Doença cerebrovascular**	1,05	0,43 - 2,52	0,925
**Insuficiência renal**	2,06	1,50 - 2,83	< 0,001
**Angioplastia prévia**	1,27	0,96 - 1,66	0,083
**Fração de ejeção (<30%)**	1,92	1,02 - 3,60	0,042
**CCS**			
IV	1,21	0,87 - 1,67	0,264
**NYHA**			
I e II	Referência	Referência	
III e IV	1,50	1,16 - 1,95	0,002
**STS score (mortalidade)**	1,38	1,25 - 1,52	< 0,001

HR: Hazard ratio; IC 95%: Intervalo de Confiança de 95%.

Tabela 2. Estimativas da associação entre características do paciente e readmissão por causas cardíacas usando um modelo de regressão univariada de Cox para a seleção de variáveis para o modelo múltiplo.

Características	HR	IC 95%	Valor de *P*
**Idade**	0,99	0,98 - 1,01	0,505
**Sexo (feminino)**	1,29	0,92 - 1,84	0,14
**Status de admissão**			
Urgência/Emergência	1,24	1,03 - 1,47	0,019
**Índice de massa corporal, kg/m²**	0,93	0,90 - 0,98	0,003
**Infarto prévio do miocárdio**	1,26	0,91 - 1,75	0,162
**Hipertensão arterial sistêmica**			

**Doença pulmonar**	0,77	0,33 - 1,77	0,544
**Tabagismo**			
Nunca	Referência	Referência	
Fumante ativo	1,05	0,66 - 1,69	0,828
Ex-fumante	1,06	0,74 - 1,5	0,752
**Diabetes mellitus**	0,96	0,70 - 1,33	0,805
**Doença cerebrovascular**	0,99	0,25 - 4,09	0,985
**Insuficiência renal**	0,94	0,48 - 1,84	0,86
**Angioplastia prévia**	1,17	0,75 - 1,83	0,485
**Fração de ejeção (<30%)**	3,53	1,65 - 7,54	0,001
**CCS**			
IV	1,41	0,86 - 2,31	0,17
**NYHA**			
I e II	Referência	Referência	
III e IV	1,23	0,79 - 1,92	0,354
**STS score (mortalidade)**	1,18	0,98 - 1,43	0,08

HR: Hazard ratio; IC 95%: Intervalo de Confiança de 95%.

Tabela 3. Causas de readmissão (considerando pacientes que readmitiram duas vezes).

Motivos de readmissão	N	%
**Angioplastia**	56	4,04
**Arritmia**	10	0,72
**Insuficiência cardíaca**	70	5,05
**Reoperação**	3	0,22
**Angina**	11	0,79
**Operação de vesícula**	16	1,15
**COVID-19**	33	2,38
**Acidente vascular cerebral**	37	2,67
**Pneumonia**	2	0,14
**Câncer**	29	2,09
**Outros**	138	9,95
