# Supplementary material for: Preditores Pré-Operatórios de Readmissão Hospitalar em até 5 Anos após CRM: Análise de Coorte do Banco de Dados REPLICCAR II
Source: Arq Bras Cardiol. 2025 Mar 6;122(2):e20240420. [Article in Portuguese] doi: 10.36660/abc.20240420 (PMC12080614; doi:10.36660/abc.20240420)
Supplement: Translation

# Translation: Preoperative Predictors of Hospital Readmission within 5 Years Following CABG: Cohort Analysis of the REPLICCAR II Database

**DOI:** 10.36660/abc.20240420i

**Authors:** Carlos Alberto Sancio, Fabiane Letícia de Freitas, Gabrielle Barbosa Borgomoni, Daniella de Lima Pes, Pedro Horigoshi Reis, Pedro Gabriel Melo de Barros e Silva, Marcelo Arruda Nakazone, Marcos Gradim Tiveron, Valquiria Pelisser Campagnucci, Luiz Augusto Lisboa, Luís Alberto Oliveira Dallan, Fabio Biscegli Jatene, Omar Asdrúbal Vilca Mejia

**Affiliations:** 1 Brazil Hospital Santa Rita de Cássia, Vitoria, ES – Brazil; 2 Brazil Instituto do Coração do Hospital das Clínicas da Faculdade de Medicina da Universidade de São Paulo, São Paulo, SP – Brazil; 3 Brazil Hospital Samaritano Paulista, São Paulo, SP – Brazil; 4 Brazil Faculdade de Medicina de São José do Rio Preto, São José do Rio Preto, SP – Brazil; 5 Brazil Irmandade da Santa Casa de Misericórdia de Marília, Marilia, SP – Brazil; 6 Brazil Faculdade de Ciências Médicas da Santa Casa de São Paulo, São Paulo, SP – Brazil

**Keywords:** Coronary Artery Disease, Myocardial Revascularization, Hospitalization

## Abstract

**Background:**

Reducing hospital readmissions following coronary artery bypass grafting (CABG) surgeries is essential to optimizing medium- and long-term patient outcomes.

**Objective:**

To analyze preoperative predictors associated with all-cause and cardiac readmissions within 5 years following CABG.

**Methods:**

We analyzed 1,387 patients who underwent CABG between June 2017 and July 2019 using data from the multicenter REPLICCAR II registry. Follow-up was carried out by telephone interviews using a questionnaire structured in the REDCap platform. Statistical analysis included univariate and multivariate methods, with Cox regression and internal validation through calibration and discrimination tests. A significance level of 5% was applied.

**Results:**

The cumulative incidence of all-cause readmission was 27.69%, with a mean follow-up of 4.3 years and a mean time to readmission of 2.4 years. Multivariate regression analysis indicated the following predictors of higher all-cause readmission risk: lower body mass index (HR=0.97, p=0.032), history of myocardial infarction (HR=1.27, p=0.024), diabetes mellitus (HR=1.35, p=0.004), renal failure (HR=1.62, p=0.004), and higher STS score (HR=1.22, p<0.001). A moderate correlation was observed between readmission and mortality (Rho=0.55).

**Conclusions:**

This analysis demonstrates that lower body mass index, history of myocardial infarction, diabetes mellitus, renal failure, and elevated STS scores are significant predictors of increased hospital readmission risk following CABG.

## Introduction

The advancement of coronary artery bypass grafting (CABG) techniques, combined with the implementation of hospital quality improvement programs, has significantly reduced postoperative morbidity and mortality rates^1 , 2^However, despite these advancements, hospital readmissions remain a challenge both in the short term^3 - 5^and long term^6 - 8^Long-term readmission is particularly difficult to define due to limited patient follow-up data.

Recent medical literature has primarily focused on hospital readmissions occurring within shorter periods after cardiac surgery, specifically within 30 or 90 days post-procedure. However, data on long-term readmissions, years after CABG, remain limited. In our setting, these results have yet to be published due to a lack of long-term records, information that is essential for defining cost-effective strategies in CABG.^9^

A subanalysis of the CORONARY trial, which studied the causes of hospital readmission within five years after CABG, identified several factors associated with an increased risk of all-cause readmission, including female sex, advanced age, high body mass index, history of acute myocardial infarction, previous cerebrovascular accident, peripheral arterial disease, active smoking, and diabetes mellitus.^7^

Identifying these risk factors can help in stratifying risk groups for prevention and improving the indication and, especially, the effectiveness of CABG surgeries.^10^This is crucial for generating value and reducing costs associated with complications in the healthcare system.^10^However, the literature shows significant variability in methodological approaches used, including differing size samples, patient allocation criteria, and outcome definitions for hospital readmission, highlighting the need for more solid evidence on this topic.

It is important to identify the factors contributing to hospital readmissions in the medium and long term to optimize cardiac surgery outcomes and minimize the financial burden on the healthcare system. Thus, the objective of this study was to identify predictors of hospital readmission for both all causes and cardiac causes within five years following CABG.

## Methods

This study is based on data from the Paulista Registry of Cardiovascular Surgery II ( *Registro Paulista de Cirurgia Cardiovascular II, REPLICCAR II* ), a prospective, multicenter study involving five hospitals in the state of São Paulo conducted with patients undergoing CABG surgery.

The REPLICCAR II database includes patients aged ≥18 years who underwent primary, isolated CABG, either electively or urgently. Data collection was conducted in the REDCap platform (http://www.project-redcap.org) specifically designed for this project, ensuring consistency with the variables and definitions from version 2.9 of the Society of Thoracic Surgeons (STS)

collection system.^11^Patients who died before hospital discharge, were readmitted within 30 days of discharge, or did not undergo isolated CABG were excluded from the analysis. Therefore, this study focuses on readmissions occurring more than 30 days after discharge.

### Patient follow-up

Follow-up data were collected between April 2023 and January 2024. Patients (N=384) answered a structured questionnaire assessing quality of life, cardiac symptoms, major cardiac adverse events, and hospital readmissions, as shown in Figure 1 .

**Figure 1:** – Flowchart analysis. CABG: coronary artery bypass grafting; REPLICCAR II: Paulista Registry of Cardiovascular Surgery. The authors adhered to the criteria established by STROCSS guidelines. ^12^

### Definition of variables

The primary outcome of this study was hospital readmission for all causes, defined as a patient’s return to the hospital within a period of up to five years following CABG.

The secondary outcome was unplanned hospital readmission due to cardiac causes, defined as heart failure, arrhythmias, angina, CABG reoperation, or percutaneous coronary intervention.

### Statistical analysis

All analyses were performed using R software, version 4.0.2.^13^The following packages were utilized for this project: *survival, car, survminer, psych, gmodels, survivalROC, timeROC, pROC, resource selection, ggplot2,* and *dplyr.*

In the descriptive analysis, continuous variables were expressed exclusively as medians and interquartile range (IQR) due to the asymmetric data distribution. Categorical variables were presented in terms of frequencies and percentages.

For the analysis of categorical independent variables, proportions were compared using the chi-square test or Fisher’s exact test, as appropriate. Data normality was assessed through the Shapiro-Wilk test. For continuous independent variables and the study outcome, the Mann-Whitney test was used to compare means since all variables presented a non-parametric distribution. To identify predictive factors, Cox univariate regression analysis was initially performed on preoperative and intraoperative variables. Variables with p-values <0.05 were subsequently included in the Cox multivariate regression model for further analysis. The findings were presented as hazard ratios (HR) with their respective 95% confidence intervals (95% CI).

To evaluate the multiple regression model, we performed the Schoenfeld residual test, suitable for Cox regression, in addition to the analysis of the Receiver Operating Characteristic (ROC) curve.

The correlation between all-cause hospital readmission and mortality was assessed using Spearman’s correlation. Rho values were interpreted as follows: Rho=0 indicates no correlation; 0 < ∣Rho∣ ≤0.3 indicates weak correlation; 0.3 < ∣Rho∣ ≤0.7 indicates moderate correlation; and ∣Rho∣ >0.7 indicates strong correlation. A significance level of 5% was adopted.

### Ethics and consent

This study is a subanalysis of the REPLICCAR II project, approved by the Ethics Committee for the Analysis of Research Projects (CAPPesq) of the Hospital das Clínicas da Universidade de São Paulo (registration number CAAE: 66919417.6.1001.0068; SDC 4506/17/006). For the follow-up phase, an amendment (opinion number 5.603.742) was approved on August 25, 2022. All patients provided consent to participate in the interview.

## Results

In the comparison between non-readmitted and readmitted patients for all causes ( Table 1 ), the readmitted group exhibited a slightly lower body mass index (p=0.022). The incidence of previous myocardial infarction (p=0.004) and the prevalence of diabetes mellitus (p=0.002) were significantly higher in this group, with a significantly higher proportion of insulin-dependent patients (p<0.001).

**Table 1:** – Preoperative characteristics of patients undergoing CABG. REPLICCAR II, São Paulo, Brazil.

Characteristics	Non-readmitted (N=1,003)	Readmitted (N=384)	p-value
**Age (years), (median and IQR)**	64 (57-70)	64 (59-71)	0.118
**Female sex, n (%)**	256 (25.52)	107 (27.86)	0.375
**Admission status, n (%)**			
Elective	574 (57.23)	198 (51.56)	
Urgency/Emergency	221 (22.03)	105 (27.34)	0.086
Transfer from another hospital	201 (20.04)	81 (21.09)	
Other	4 (0.40)	0 (0.00)	
**Body mass index, kg/m^2^(median and IQR)**	27 (24.50-29.76)	26.57 (24.22-29.38)	0.022
**Previous myocardial infarction, n (%)**	495 (49.35)	223 (58.07)	0.004
**Systemic arterial hypertension, n (%)**	880 (87.74)	343 (89.32)	0.413
**Pulmonary disease, n (%)**			
Mild	10 (1.00)	6 (1.56)	0.321
Moderate	4 (0.40)	3 (0.78)	
Severe	5 (0.50)	0 (0.00)	
**Smoking, n (%)**			
Non-smoker	509 (50.75)	185 (48.16)	0.549
Active smoker	146 (14.56)	64 (16.67)	
Ex-smoker	348 (34.70)	135 (35.16)	
**Diabetes mellitus (n, %)**	484 (48.26)	221 (57.55)	0.002
Insulin-dependent	114 (11.37)	71 (18.49)	< 0.001
**Cerebrovascular disease, n (%)**	79 (7.88)	52 (13.54)	0.001
**Renal failure, n (%)**			
Chronic	42 (4.19)	40 (10.42)	<0.001
Acute	7 (0.70)	3 (0.78)	
**Creatinine, mg/dL (median and IQR)**	1.07 (0.93-1.37)	1.16 (0.90-1.23)	< 0.001
**Previous angioplasty, n (%)**	134 (13.36)	63 (16.41)	0.145
**Ejection fraction (<30%), n (%)**	11 (1.10)	10 (2.60)	0.039
**CCS, n (%)**			
IV	89 (8.87)	40 (10.42)	0.376
**NYHA, n (%)**			
I and II	891 (88.83)	316	0.006
III and IV	112 (11.17)	68	
**STS mortality score (median and IQR)**	0.81 (0.43-0.97)	1.05	<0.001

CCS: Canadian Cardiovascular Society angina grading; IQR: interquartile range; NYHA: New York Heart Association functional classification; STS: Society of Thoracic Surgeons. Cerebrovascular disease: stroke, transient ischemic attack, or carotid stenosis ≥50%; renal failure: creatinine clearance <60 mL/min/1.73 m^2^.

The readmitted group also had a higher frequency of cerebrovascular disease (p=0.001) and renal failure (p<0.001), along with elevated creatinine levels (p<0.001). Cardiac function analysis revealed that an ejection fraction below 30% was more common in the readmitted group (p=0.039).

In terms of functional status, New York Heart Association (NYHA) classifications III and IV were more prevalent in the readmitted group (p=0.006), suggesting a greater heart failure severity. Finally, the STS score for mortality was significantly higher in the readmitted group (p<0.001).

Significant differences were observed between non-readmitted and readmitted patients regarding the use of cardiopulmonary bypass (CPB) and extubation in the operating room (p=0.028 and 0.040, respectively). Furthermore, blood glucose levels were significantly higher (p<0.001) in the readmitted patients, as shown in Table 2 .

**Table 2:** – Intraoperative characteristics of patients undergoing CABG. REPLICCAR II, São Paulo, Brazil

Characteristics	Non-readmitted (N=1,003)	Readmitted (N=384)	p-value
**CPB use, n (%)**	901 (89.93)	328 (85.42)	0.028
**CPB time (min), (median and IQR)**	75 (59.00-95.25)	75 (56-100)	0.998
**Aortic clamping time (min), (median and IQR)**	57 (43-75)	59 (41-79)	0.516
**Use of bilateral thoracic artery, n (%)**	135 (13.46)	45 (11.72)	0.388
**Use of left internal thoracic artery, n (%)**	966 (96.31)	361 (94.01)	0.059
Pedicled	629 (65.11)	243 (67.31)	0.128
Skeletonized	337 (34.89)	118 (32.69)	
**Use of right internal thoracic artery, n (%)**	145 (14.46)	51 (13.28)	0.573
Pedicled	75 (51.72)	27 (52.94)	0.844
Skeletonized	70 (48.28)	24 (47.06)	
**Use of radial artery, n (%)**	46 (4.59)	18 (4.69)	0.935
**Duration of surgery (h), (median and IQR)**	4.50 (3.42-6.00)	4.67 (3.50-6.08)	0.314
**Packed red blood cell transfusion, n (%)**	8 (0.80)	3 (0.78)	0.970
**Extubation in operating room, n (%)**	48 (4.79)	9 (2.34)	0.040
**Higher blood glucose (median and IQR)**	174 (47.17-93.08)	177 (50.50-115.25)	< 0.001

CPB: cardiopulmonary bypass; IQR: interquartile range.

As shown in Table 3 , readmitted patients had a higher incidence of renal failure (p<0.001), an increased rate of reoperation for bleeding (p=0.009), and longer durations of orotracheal intubation (p=0.001). Prolonged ventilation exceeding 24 hours (p<0.001) and surgical wound infection (p=0.013) were also more common in this group. Additionally, readmitted patients experienced longer hospital stays (p<0.001) and intensive care unit (ICU) stays (p<0.001), including hospitalizations exceeding 14 days (p<0.001), highlighting the complexities of postoperative management in these cases.

**Table 3:** – Postoperative outcomes and evolution of patients undergoing CABG. REPLICCAR II, São Paulo, Brazil

Characteristics	Non-readmitted (N=1,003)	Readmitted (N=384)	p-value
**Cerebrovascular accident, n (%)**	13 (1.30)	5 (1.30)	0.992
**Renal failure, n (%)**	45 (4.49)	46 (11.98)	< 0.001
**Reoperation for bleeding, n (%)**	2 (0.20)	5 (1.30)	0.009
**Atrial fibrillation, n (%)**	143 (14.26)	68 (17.71)	0.109
**Orotracheal intubation time (h), (median and IQR)**	7.78 (5.33-11.01)	8.5 (6.04-12.23)	0.001
**Prolonged ventilation (>24 h), n (%)**	25 (2.49)	24 (6.25)	< 0.001
**Surgical wound infection (≤30 days), n (%)**	22 (2.19)	18 (4.69)	0.013
**Prolonged hospital stay (>14 days), n (%)**	222 (22.13)	119 (30.99)	< 0.001
**Short hospital stay (<6 days), n (%)**	52 (5.18)	13 (3.39)	0.156
**Length of ICU stay (hours), (median and IQR)**	68.12 (47.17-93.08)	73.17 (50.50-115.25)	< 0.001
**Total length of hospital stay (days) (median and IQR)**	7.00 (6.00-8.00)	7.00 (6.00-10.00)	< 0.001
**30-day post-procedure mortality, n (%)**	0 (0.00)	5 (1.30)	0.002

ICU: intensive care unit; IQR: interquartile range.

To identify factors associated with all-cause readmission, univariate regression was used to determine variables correlated with the event ( Supplementary Table 1 ). These variables were subsequently included in a multivariate regression to create a multiple model ( Table 4 ).

**Table 4:** – Multivariate Cox regression model: association between patient characteristics and all-cause readmission (adjusted for patient characteristics). REPLICCAR II, São Paulo, Brazil

Characteristics	HR	95% CI	p-value
**Admission status**			
Urgency	1.11	0.99 - 1.24	0.070
**Body mass index, kg/m^2^**	0.97	0.94 - 0.99	0.029
**Previous myocardial infarction**	1.27	1.03 - 1.56	0.024
**Diabetes mellitus**	1.35	1.10 - 1.66	0.004
Renal failure*	1.62	1.16 - 2.25	0.004
**Ejection fraction (<30%)**	1.32	0.70 - 2.50	0.394
**NYHA**			
III and IV	1.28	0.97 - 1.67	0.077
**STS score (mortality)**	1.22	1.09 - 1.36	< 0.001

95% CI: 95% confidence interval; HR: hazard ratio; NYHA: New York Heart Association functional classification; STS: Society of Thoracic Surgeons. * Creatinine clearance <60 mL/min/1.73 m^2^.

To validate the model, the Schoenfeld residuals test was performed, and the results showed no significant evidence of proportional hazard assumption violations, indicating proper model calibration (p=0.192). Furthermore, the ROC curve (AUC 0.80, 95% CI 0.76-0.84) demonstrated that the multiple model is accurate in predicting all-cause readmission within 5 years after CABG ( Figure 2 and central figure ).

**Figure 2:** – ROC curve of the multivariate Cox regression model adjusted for patient characteristics predicting all-cause readmission.

To identify factors associated with all-cause readmission, univariate regression was used to determine variables correlated with the event ( Supplementary Table 2 ). These variables were then incorporated into a multivariate regression analysis to create a multiple model ( Table 5 ).

**Table 5:** – Multivariate Cox regression model: association between patient characteristics and all-cause readmission (adjusted for patient characteristics). REPLICCAR II, São Paulo, Brazil

Characteristics	HR	95% CI	p-value
**Admission status**			
Urgency	1,22	1,02 - 1,46	0,026
**Body mass index, kg/m^2^**	0,94	0,90 - 0,98	0,005
**Ejection fraction (<30%)**	3,15	1,47 - 6,76	0,003

95% CI: 95% confidence interval; HR: hazard ratio.

To validate the model, the Schoenfeld residuals test was performed, and the results showed no significant evidence of proportional hazard assumption violations, indicating proper model calibration (p=0.192). In addition, the ROC curve (AUC 0.76, 95% CI 0.73-0.79) ( Figure 3 ) demonstrated that the multiple model is accurate to predict cardiac-related readmission within 5 years after CABG.

**Figure 3:** – ROC curve of the multivariate Cox regression model adjusted for predicting cardiac-related readmission.

The mean follow-up time for patients was 4.3 years (25^th^and 75^th^percentiles: 3.5-5.0). The cumulative incidence of all-cause readmission was 27.69% (95% CI 0.25-0.30), as shown in Figure 4-A . The mean time to all-cause readmission was 2.4 years (25^th^and 75^th^percentiles: 1.0-3.6). For cardiac-related readmissions, the cumulative incidence was 10.67%. Regarding readmission due to cardiac causes ( Figure 4-B ), the mean time was 2.33 years (25^th^and 75^th^percentiles: 0.75-3.69).

Among patients who were not readmitted, only 15 (1.50%) died. In contrast, 154 (40.10%) of readmitted patients died. A moderate correlation was observed between all-cause hospital readmission and mortality, with a Rho coefficient of 0.55 (95% CI 0.51-0.59).

**Figure 4:** – Cumulative incidence of all-cause (A) and cardiac-related (B) readmissions in the cohort during follow-up.

## Discussion

This analysis of medium- and long-term follow-up in patients undergoing CABG in the state of São Paulo, based on multicenter data, revealed that the cumulative incidence of cardiac-related readmission was 10.67%, while all-cause readmission was 27.69%. The latter was found to be correlated with mortality, consistent with current literature.^7^

Among the identified predictors, urgent surgical status, reduced body mass index (BMI), and left ventricular ejection fraction (LVEF) <30% were significant for both outcomes evaluated, highlighting the complexity of patients in this cohort.

Although mortality rates in cardiac surgery have significantly declined due to advancements in surgical techniques, equipment, care management, databases, and others,^2 , 14 , 15^attention has increasingly shifted toward non-mortality outcomes, particularly in discussions about patient quality and safety. However, such data remain scarce and fragmented in low- and middle-income countries, especially regarding long-term follow-up. Most analyses focus on the first 30 to 90 days after discharge, with reported readmission rates ranging from 8.3 and 21.1%, reflecting substantial variability across studies.^4 , 16 , 17^

The lack of large, well-structured health databases for long-term follow-up in developing countries not only limits the ability to conduct robust research and develop data-driven strategies but also compromises the follow-up of patients undergoing CABG. Without a detailed record, with periodic updates, medical histories of diagnoses, and interventions, health professionals, hospital administrators, and public health officials face obstacles in tracking patient progress, identifying postoperative complications, adapting care plans, and ensuring optimal patient outcomes. Implementing user-friendly and robust data systems is essential for fostering a data-driven culture, improving healthcare delivery, and enabling faster disease identification, optimal treatment selection, and effective monitoring of outcomes.^18^Hospital readmissions, for example, represent an outcome linked to poorer prognosis and increased healthcare costs.^19 , 20^

In our analysis, 40.10% of patients who required at least one readmission after hospital discharge following CABG progressed to death. Similarly, Bianco et al.^21^evaluated the long-term impacts of readmission within 30 days after surgery (N=14,538), demonstrating that readmission was significantly associated with mortality both at short (6 months) and long follow-up (60 months). It also emerged as an independent predictor of subsequent readmissions. Further studies are needed to assess the quality of life in patients who require new hospitalizations after cardiac procedures. This assessment should consider not only clinical factors but also psychosocial aspects, allowing hospitals to develop appropriate follow-up tools and strategies to reduce readmission rates.

In this context, the creation of predictive models and risk scores emerges as a powerful tool to support medical and multiprofessional decision-making, with the potential to reduce the risk of readmission significantly. The factors associated with readmission through multivariate regression reflect the complexity of preoperative patient conditions, providing opportunities for improvement. These findings align with prior literature,^6^including the CORONARY trial,^6^which evaluated 4,623 patients with long-term follow-up. The trial highlighted the substantial rates of all-cause and cardiac-related readmissions within five years post-CABG and revealed a strong correlation between readmission and long-term mortality. The study also showed that women had a significantly higher risk of readmission, with a strong correlation between readmission and long-term mortality, showing significant gender differences in prognosis after the procedure. In contrast, our study did not find sex to be significantly associated with any of the outcomes evaluated; however, we observed a moderate correlation between all-cause readmission and mortality. More studies need to be carried out to explore the impact of sex in the progression and prognosis of patients undergoing CABG.^22^

In our analysis, low BMI was a predictor of long-term hospital readmission. Malnutrition, closely correlated with readmission, may reflect delays in performing cardiac surgery due to the considerable surgical queue in the public health system. These delays often occur without adequate multidisciplinary follow-up or structured prehabilitation programs. Additionally, it may be associated with weight loss during pre-surgical hospitalization, as sarcopenia is well-documented to correlate with poorer clinical outcomes.^23^This finding underscores the importance of nutritional assessment and management of the patient’s weight at the time of surgical indication, preparing them for surgery based on personalized and preventive care management as strategies to optimize the patient’s clinical status at the time of surgery, promoting a more efficient recovery and with the potential to minimize the risk of readmission.

Heart failure is widely recognized as a condition associated with multiple hospitalizations and worsening morbidity and mortality rates.^24 - 26^The identification of LVEF <30% as a predictor of hospital readmission is reasonable and can be explained by delays in diagnosing cardiovascular disease or prolonged waiting periods for surgical treatment, which is often performed under urgent circumstances.^27^An LVEF <30% contributes to the deterioration of a patient’s clinical condition, leading to decompensations that require additional hospitalizations.

The use of CPB was less common among readmitted patients.

Studies suggest that CPB may increase the risk of complications due to the systemic inflammatory response and mechanical trauma associated with the procedure.^28^However, since this study was not randomized, it is possible that patients who did not undergo CPB had a more severe clinical profile, such as porcelain aorta or other unmeasured risk factors, which could have influenced the different complication profiles observed. Although the literature suggests that off-pump surgeries can reduce immediate postoperative complications, there is still no consensus on the long-term impact.^29^

Extubation in the operating room was less common among readmitted patients. This practice is generally associated with better outcomes, such as reduced mechanical ventilation, ICU time, and length of hospital stay.^30 , 31^The literature indicates that early extubation when performed in carefully selected patients, may decrease the risk of pulmonary infections and promote faster postoperative recovery, which may contribute to a reduced rate of readmissions.

Studies have shown that tight glycemic control can reduce the risk of postoperative complications and hospital readmissions, especially in diabetic patients. In our study, readmitted patients had higher glycemic levels compared to non-readmitted patients. Perioperative hyperglycemia is associated with worse outcomes, including increased infectious complications, delayed wound healing, and higher mortality. These findings align with the literature, which highlights the importance of maintaining adequate glycemic control during the intraoperative period, regardless of whether the patient has diabetes.^32 , 33^

Long-term readmitted patients exhibited hospital characteristics associated with worse outcomes, such as renal failure, higher rate of reoperations for bleeding, and deep sternal wound infection/mediastinitis. These findings indicate that postoperative complications associated with longer hospital and ICU stays are related to an increased risk of long-term readmissions. The use of performance indicators to evaluate clinical practice is fundamental for effective hospital management. Hospital readmission not only reflects the quality of care and patient satisfaction but also allows monitoring processes that can lead to readmissions. Such events postpone the patient’s return to daily activities, expose them again to additional hospital risks, and potentially increase the risk of complications and death. In our analysis, we highlighted the importance of this indicator, observing a moderate correlation between all-cause readmission and mortality, with a Rho coefficient of 0.55 (95% CI 0.51-0.59) ( Figure 4 ). Identifying and monitoring these indicators is crucial for the development of proactive strategies aimed at improving care outcomes and reducing costs.^20^

One of the main challenges of this study was the follow-up of patients, which required telephone calls. Brazil still lacks a single database system that centralizes information on readmissions and deaths, which could facilitate and advance national research efforts. Without effective data integration, achieving comprehensive and accurate patient follow-up remains a challenge. The absence of a centralized database complicates data collection and analysis, both of which are essential for the continuous improvement of healthcare quality and management.

We believe that implementing a robust patient follow-up database has the potential to identify predictors and guide interventions, improving surgical and medical care outcomes and enhancing patients’ quality of life.

### Study limitations

This study was conducted with a preliminary cohort from a multicenter database in the state of São Paulo. Follow-up efforts are ongoing, with the study group attempting contact (up to five attempts) for each patient included in the primary database. However, the difficulty in locating a significant proportion of the patients included initially in the study may compromise the accuracy and generalization of the results obtained. Institutional-specific results may have influenced the analysis due to heterogeneity in health team expertise, available resources, institutional methodologies, protocols, and patient profiles. Therefore, it is recommended that each institution evaluate its own data independently to validate the results presented here and guide strategies for improving outcomes.

This analysis did not focus on complications occurring within the first 30 days after CABG, as the study aimed to examine long-term outcomes among surgery survivors to identify preoperative predictors of hospital readmission and develop strategies to reduce this impact. This is a post-hoc analysis, as long-term follow-up was not the main objective of the REPLICCAR II database. As such, the statistical power of this analysis may be insufficient to validate the hypotheses presented fully. Further studies are recommended to confirm these findings.

## Conclusion

The preoperative variables, low BMI, history of myocardial infarction, diabetes mellitus, renal failure, and a high STS score were associated with an increased risk of hospital readmission within 5 years after CABG. Likewise, emergency surgery, low BMI, and an ejection fraction <30% were predictors of readmissions due to cardiac causes.
